# Oligodendrocyte‐derived exosomes‐containing SIRT2 ameliorates depressive‐like behaviors and restores hippocampal neurogenesis and synaptic plasticity via the AKT/GSK‐3β pathway in depressed mice

**DOI:** 10.1111/cns.14661

**Published:** 2024-03-04

**Authors:** Honghan Zhang, Xin‐hui Xie, Shu‐xian Xu, Chao Wang, Siqi Sun, Xinhua Song, Ruiling Li, Ningyuan Li, Yuqi Feng, Hao Duan, Di Li, Zhongchun Liu

**Affiliations:** ^1^ Department of Psychiatry Renmin Hospital of Wuhan University Wuhan Hubei China; ^2^ Clinical College of Traditional Chinese Medicine Hubei University of Chinese Medicine Wuhan China; ^3^ Department of Laboratory Medicine, Tongji Hospital, Tongji Medical College Huazhong University of Science and Technology Wuhan China; ^4^ Taikang Center for Life and Medical Sciences Wuhan University Wuhan China

**Keywords:** depression, neurogenesis, oligodendrocyte‐derived exosome, sirtuin 2, synaptic plasticity

## Abstract

**Aims:**

To investigate the antidepressant role of oligodendrocyte‐derived exosomes (ODEXs)‐containing sirtuin 2 (SIRT2) and the underlying mechanism both in vivo and in vitro.

**Methods:**

Oligodendrocyte‐derived exosomes isolated from mouse serum were administered to mice with chronic unpredictable mild stress (CUMS)‐induced depression via the tail vein. The antidepressant effects of ODEXs were assessed through behavioral tests and quantification of alterations in hippocampal neuroplasticity. The role of SIRT2 was confirmed using the selective inhibitor AK‐7. Neural stem/progenitor cells (NSPCs) were used to further validate the impact of overexpressed SIRT2 and ODEXs on neurogenesis and synapse formation in vitro.

**Results:**

Oligodendrocyte‐derived exosome treatment alleviated depressive‐like behaviors and restored neurogenesis and synaptic plasticity in CUMS mice. SIRT2 was enriched in ODEXs, and blocking SIRT2 with AK‐7 reversed the antidepressant effects of ODEXs. SIRT2 overexpression was sufficient to enhance neurogenesis and synaptic protein expression. Mechanistically, ODEXs mediated transcellular delivery of SIRT2, targeting AKT deacetylation and AKT/GSK‐3β signaling to regulate neuroplasticity.

**Conclusion:**

This study establishes how ODEXs improve depressive‐like behaviors and hippocampal neuroplasticity and might provide a promising therapeutic approach for depression.

## Graphical abstract

We investigated the antidepressant role of oligodendrocyte‐derived exosomes (ODEXs)‐containing SIRT2 both in vivo and in vitro. ODEXs reversed CUMS‐induced depressive‐like behaviors and impairments in neurogenesis and synaptic plasticity. Mechanistically, ODEXs mediated transcellular delivery of SIRT2, targeting AKT deacetylation and AKT/GSK‐3β signaling to regulate neuroplasticity.

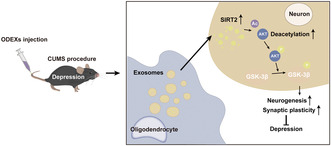

## 1 INTRODUCTION

Major depressive disorder (MDD) is a common but heterogeneous neuropsychiatric disorder characterized by persistent low mood and is associated with high suicide rates.^1^ Despite progress in treatments for MDD, current antidepressants typically require several weeks to take effect and only benefit about a third to half of individuals with depression.^2^ To improve the treatment of depression, it is essential to understand the underlying molecular mechanisms to inform therapy development.^3^

Hippocampal neurogenesis and synaptic plasticity are vital indicators of neuroplasticity, which describes the brain's capacity to adapt to internal and external stimuli.^4^, ^5^ Depressed patients and animal models of depression show features of impaired neurogenesis and synaptic dysfunction.^6^, ^7^, ^8^, ^9^ Since some antidepressants work by enhancing neurogenesis and synaptic plasticity, investigating the modulation of neuroplasticity as a therapeutic target could be of clinical benefit.^5^

Exosomes are small, double‐membraned vesicles that envelope various biomolecular cargoes.^10^ In the central nervous system (CNS), exosomes can regulate intercellular communication between glial cells and neurons to modulate neuroplasticity.^11^, ^12^ A recent study showed that exosomes secreted by microglia can upregulate and deliver microRNAs (miRNAs) to inhibit neurogenesis in the hippocampal dentate gyrus (DG), consequently exacerbating depressive‐like behaviors in rats subjected to chronic unpredictable mild stress (CUMS).^13^

Recent studies have highlighted a pivotal role for oligodendrocytes in the pathobiology of depression, given their critical functions in myelin sheath formation, support of axonal energy metabolism, and regulation of various neuroplasticity processes.^14^, ^15^ Oligodendrocytes can secrete exosomes and deliver specific cargoes to neurons, thereby contributing to the preservation of neuronal structure and function.^16^, ^17^, ^18^ In addition, oligodendrocyte‐derived exosomes (ODEXs) carrying specific proteins can enhance the stress resilience of neurons.^19^, ^20^ Nevertheless, the effects of these exosomes and their cargoes on depression remain unknown.

Sirtuin 2 (SIRT2) is a brain‐enriched NAD‐dependent deacetylase.^21^ In the sirtuin family, SIRT2 stands out as being predominantly cytoplasmic and much more abundantly expressed in oligodendrocytes than in neurons.^22^ The transcellular delivery of this protein from oligodendrocytes to neurons via exosomes plays a vital role in CNS function.^16^ SIRT2 is implicated in neuropsychiatric disorders,^23^ and clinical studies have indicated that SIRT2 transcript is reduced in peripheral leukocytes and hippocampal tissues of depressed patients.^24^, ^25^ In rodents, reduced hippocampal SIRT2 is associated with the progression of depression and impaired neuroplasticity, while SIRT2 overexpression can enhance hippocampal neurogenesis and alleviate depressive symptoms.^26^, ^27^ However, there have been no studies on the biological role of exosomal SIRT2 in depression.

To address this gap, here we explored the antidepressant role of ODEXs in hippocampal neurogenesis, synaptic plasticity, and depressive‐like behaviors in CUMS mice. Furthermore, we investigated the capacity of ODEXs to deliver SIRT2 cargo to neurons for the regulation of neuroplasticity via the AKT/GSK‐3β pathway. In addition, the impact of overexpressed SIRT2 and ODEXs on neurogenesis and synapse formation was corroborated in neural stem/progenitor cells (NSPCs).

## 2 MATERIALS AND METHODS

### 2.1 Experimental animals and groups

Male C57BL/6J mice (8‐ to 10‐week‐old) were purchased from the Hunan Slack King Laboratory Animal Co. (Changsha, Hunan, China). All mice were housed in controlled environments. The Ethical and Animal Welfare Committee of Wuhan University approved all animal experiments. Mice were randomly divided into a control group (Control), CUMS group (CUMS), CUMS + ODEX group (ODEXs), and CUMS + ODEX + AK‐7 group (ODEXs + AK‐7). After 4 weeks of the CUMS procedure, mice were injected with 100 μL of ODEXs (0.5 μg/μL) or an equal volume of PBS via the tail vein every 3 days for another 2 weeks—a total of five injections. To explore the role of SIRT2, mice were intraperitoneally injected with a SIRT2 inhibitor, AK‐7 (20 mg/kg), or an equal volume of PBS once per day over the last 2 weeks of the CUMS procedure.

### 2.2 CUMS model

Chronic unpredictable mild stress was conducted as previously with minor modifications.^2^ In brief, mice were subjected to two random mild stressors each day for 6 weeks. These stressors included 24‐h food deprivation, 24‐h water deprivation, 24‐h cage tilting at 45°, 24‐h damp bedding, 24‐h solid cage, 24‐h light/dark cycle reversal, 6‐h physical restraint, 30‐min cage shaking at 120 rpm, 10‐min tail pinching, and 5‐min swimming in 4°C water. The specific schedule for CUMS is listed in Table S1.

### 2.3 Behavioral tests

#### 2.3.1 Sucrose preference test (SPT)

Mice were provided with two identical water bottles. For the first 24 h, both bottles contained water, and in the following 24 h, they were filled with 1% sucrose solution. Water was then removed for the next 24 h. During the test, one bottle contained water, while the other contained 1% sucrose solution. The positions of the two bottles were swapped in the middle of the test. The sucrose preference index was calculated as the ratio of the amount of 1% sucrose solution consumed by mice to their total liquid consumption.

#### 2.3.2 Open field test (OFT)

Mice were placed in the experimental room 3 h before the test. During the test, mice were positioned in the center of an open field apparatus (50 × 50 × 35 cm), and their movements were recorded for 5 min. The total distance and the time in the center region were analyzed with a video‐tracking system (EthoVision XT 11.5, Noldus, Wageningen, the Netherlands).

#### 2.3.3 Forced swimming test (FST)

Mice were placed in a glass cylinder (diameter 15 cm, height 30 cm) filled with water (depth 15 cm, temperature 24 ± 1°C) and subjected to forced swimming for 6 min. Total immobility time during the last 4 min was calculated.

### 2.4 Isolation and identification of ODEXs

Oligodendrocyte‐derived exosomes were isolated using a two‐step immunoprecipitation‐based method for extracting brain‐derived exosomes from blood, which has been widely tested and well verified.^28^, ^29^, ^30^ Briefly, 250 μL of mouse serum was mixed with Dulbecco's phosphate‐buffered saline (DPBS, Absin, abs970, China) and centrifuged at 3000 × *g* for 10 min at 4°C to remove cells and cell debris. Subsequently, 126 μL of ExoQuick (System Biosciences, EXOQ20A‐1, Palo Alto, CA) was added to the supernatant and incubated for 60 min at 4°C and then centrifuged at 1500 × *g* for 30 min to obtain total exosomes (TEXs). The pellets were resuspended in 350 μL of DPBS and incubated with 2 μL of anti‐CNPase biotinylated antibody (G‐Biosciences, ITT1003‐100u‐B, St Louis, MO) and 50 μL of 3% bovine serum albumin (BSA, Beyotime, ST023‐50g, Shanghai, China) for 1 h at room temperature (RT). Ten microliter of streptavidin agarose resin (Thermo Fisher Scientific, 53116, Waltham, MA) and 40 μL of 3% BSA were added to the solution and incubated for 30 min at RT and then centrifuged at 800 × *g* for 10 min. One‐hundred microliter of 0.05 M glycine–HCl (pH = 3.0) was added to resuspend the pellets and then centrifuged at 4000 × *g* for 10 min at 4°C. The supernatant enriched with CNPase‐positive exosomes was collected, and 10 μL of 1 M Tris–HCl (pH = 8.0) was added to adjust the pH. These obtained CNPase‐positive sub‐populations constituted the final ODEX samples, which were stored to facilitate subsequent experiments.^31^ Exosome morphology was confirmed by transmission electron microscopy (TEM; Hitachi, HT7800, Tokyo, Japan). The concentration and particle size distribution of exosomes were measured by nanoparticle‐tracking analysis (NTA; Particle Metrix, ZetaView PMX 110). The presence of three exosomal markers (CD9, CD63, and Alix) and an oligodendrocyte marker (MBP) was verified by western blotting.

### 2.5 ODEX uptake

Oligodendrocyte‐derived exosomes were labeled with PKH26 dye (Sigma‐Aldrich, MINI26, St Louis, MO). Labeled exosomes were co‐cultured with NSPCs for 24 h, and ODEX uptake in vitro was observed by fluorescence microscope (Olympus, BX51). Mice were injected with the labeled exosomes via the tail vein and perfused after 6 h. Brain sections were stained with anti‐MAP2 antibodies and DAPI to verify that neurons can take up ODEXs in vivo.

### 2.6 Cell culture and administration

Neural stem/progenitor cells were derived from E14.5 mouse embryonic cortex and hippocampus according to reported protocols.^32^, ^33^ NSPCs were cultured in DMEM/F12 medium supplemented with B27 supplement, GlutaMax, heparin, 20 ng/mL bFGF, 20 ng/mL EGF, and streptomycin/penicillin. For neural differentiation, NSPCs were plated on poly‐L‐lysine co‐coated 12‐well plates at the density of 1.5 × 10^5^/mL and cultured in DMEM/F12 medium supplemented with B27 supplement, glutamine, heparin, 2% FBS, and streptomycin/penicillin for 14 days. The medium was replaced once every 3 days, and cells were cultured in a humidified incubator at 37°C with 5% CO_2_. After 12 h, NSPCs in the ODEX group were incubated with ODEXs (10 μg/mL), and cells in the ODEX + AK‐7 group were incubated with ODEXs (10 μg/mL) and AK‐7 (10 μM). The treatment was administered every 3 days with regular medium changes.

### 2.7 Lentivirus production and infection

Mouse *SIRT2* cDNA construct was cloned into the VP032‐EF1‐MCS‐3flag‐EF1‐ZsGreen‐T2A‐PURO vector. HEK293T cells were transfected with vectors with pMD.2G/psPAX2 plasmids, and mature lentivirus was obtained by ultracentrifugation. NSPCs were transfected with the vector or SIRT2 lentivirus after 12 h of inoculation, and the medium containing lentivirus was refreshed after 24 h.

### 2.8 Western blotting

Proteins were extracted from hippocampal tissues, NSPCs, and exosomes. Target proteins were separated by 10%–12% SDS‐PAGE gels and transferred to PVDF membranes. Five percent non‐fat milk was used to block non‐specific binding, and the primary antibodies were incubated at 4°C overnight. After incubation with the secondary antibodies at RT for 1 h, protein bands were visualized with a ChemiDoc Imaging System (Bio‐Rad, Hercules, CA) and quantified with ImageJ software. The antibodies are listed in Table S2.

### 2.9 Immunofluorescence staining

Brain tissues were fixed, embedded in paraffin, and sectioned into 4 μm slices. After dewaxing and rehydration, antigens were retrieved. Sections were blocked with 10% BSA and incubated with primary antibodies overnight. After incubation with the secondary antibodies at RT for 1 h, sections were stained with DAPI for 5 min.

Cells were fixed in 4% paraformaldehyde for 30 min and permeabilized with 0.1% Triton X‐100. The following procedure was identical to that used for the brain sections, as described above. Images were obtained using a fluorescence microscope (Olympus, BX51). The antibodies are listed in Table S3.

The number of DCX^+^ and Nestin^+^ cells in the DG region was counted using ImageJ software. The results were normalized to the area of DG per mm^2^ in each section, with analyses conducted on three mice per group. To quantify the percentage of DCX^+^ cells in vitro, three slides per group were analyzed to calculate the positive cell number by ImageJ software. The number of positive cells was normalized to DAPI.

### 2.10 Real‐time PCR (RT‐PCR)

Total RNA from the hippocampal tissues and NSPCs was extracted with TRIzol reagent (Servicebio, G3013, Hubei, China). cDNA synthesis was performed using all‐in‐one RT EasyMix for qPCR (Tolobio, 22107, Shanghai, China). RT‐PCR reactions were performed using SYBR qPCR Master Mix (Tolobio, 22204). Relative expression was calculated by the standard 2^−ΔΔCt^ method. The primer sequences are listed in Table S4.

### 2.11 Transmission electron microscopy (TEM)

Hippocampal DG tissues were carefully dissected into 1 mm^3^ segments and then fixed in 2.5% glutaraldehyde followed by 1% osmium acid. After dehydration in ethanol, these segments were immersed in a semi‐epoxy‐propane mixture overnight, embedded in resin, and cut into 70 nm sections. Ultrathin sections were stained on the copper grid with 4% uranyl acetate and 0.5% lead citrate. The morphology of hippocampal synapses was observed by TEM (Hitachi, HT7700).

For the analysis of synaptic density, only structures with clear synaptic clefts and vesicles were counted as synapses, and the number of synapses was divided by per μm^2^ in the DG region.

### 2.12 Golgi‐Cox staining

Hippocampal tissue blocks were completely immersed in Golgi dye solution (Servicebio, G1069) for 14 days, with the staining solution refreshed every 3 days. Following immersion in 80% glacial acetic acid, blocks were dehydrated in 30% sucrose and then sectioned into 100 μm slices. After treatment with ammonia and acid‐hardening fixing solution, sections were sealed using glycerin gelatin.

For quantification of spine density in the DG region, the slides were imaged using an electron microscope (Nikon E100, Japan), which provides adequate resolution for counting spines. All dendrites were randomly selected from distal regions for analysis. The length of dendrites and the number of dendritic spines were measured using Fiji ImageJ software, which has previously been shown to be equivalent to manual counting.^34^, ^35^

### 2.13 Immunoprecipitation assay

Cells were lysed with immunoprecipitation lysis buffer (Beyotime, P0013). Five‐hundred microgram protein was incubated with 1 μg anti‐AKT (Cell Signaling Technology, 9272S, Danvers, MA) or anti‐IgG (Beyotime, A7016) overnight at 4°C, followed by a 2‐h incubation with 20 μL protein A + G agarose (Beyotime, P2019) at 4°C. The immunoprecipitates were thoroughly resuspended by vortexing and were separated by SDS‐PAGE for immunoblotting analysis.

### 2.14 Statistical analysis

Statistical analysis was conducted using GraphPad Prism v9.0.0 (GraphPad Software, La Jolla, CA). All data were tested for normality by the Shapiro–Wilk test. Data analysis between two groups was carried out using unpaired Student's *t*‐tests. Data were compared between >2 groups by one‐way ANOVA with Tukey's multiple‐comparisons test. A *p*‐value < 0.05 was considered significant.

## 3 RESULTS

### 3.1 ODEX characterization and uptake

**FIGURE 1:**
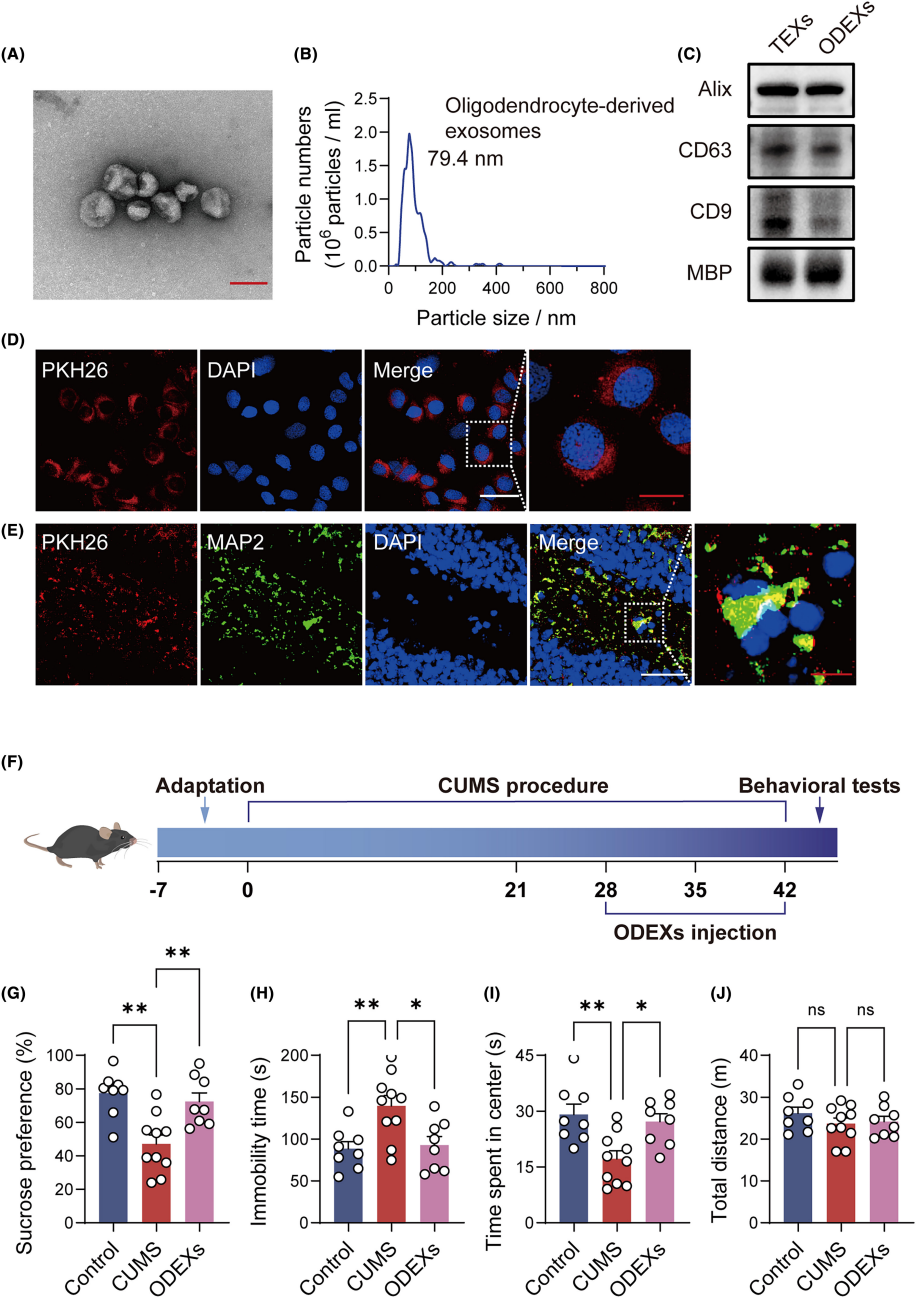
ODEX characterization and uptake and their effects on CUMS‐induced depressive‐like behaviors in mice. (A) TEM image of ODEXs isolated from mouse serum. Scale bar, 100 nm. (B) The size distribution of ODEXs. (C) Western blotting analysis of CD9, CD63, Alix, and MBP in TEXs and ODEXs. Full‐unedited gels are presented in Figure S1. (D) Representative images of PKH26‐labeled ODEXs internalized by NSPCs. Scale bars, 50 μm (white) and 20 μm (red). (E) Representative images showing ODEX uptake into hippocampal neurons. Scale bars, 50 μm (white) and 10 μm (red). (F) Schematic of the animal experiments. (G–J) Effects of ODEXs on sucrose preference in the SPT, immobility time in the FST, and center time and traveled distance in the OFT (*n* = 8–10). Data are expressed as mean ± SEM. ns, not significant, *p < 0.05, and **p < 0.01 by one‐way ANOVA with Tukey's multiple‐comparisons test.

Oligodendrocyte‐derived exosomes were extracted from normal mouse serum. TEM images showed the typical round or oval shape of exosomes (Figure 1A). NTA confirmed that ODEXs had a homogeneous particle size concentrated at 79.4 nm, consistent with the expected particle size distribution of exosomes (Figure 1B). Exosomal markers CD9, CD63, Alix, and an oligodendrocyte marker MBP were all identified by western blotting (Figure 1C). Hence, these isolated samples were verified to be ODEXs.

To validate ODEX uptake, we labeled ODEXs with PKH26. Dye‐labeled ODEXs were cultured with NSPCs and intravenously injected into mice. Uptake was confirmed using immunofluorescence staining both in vitro and in vivo (Figure 1D,E).

### 3.2 ODEXs significantly ameliorate depressive‐like behaviors in CUMS mice

Three common behavioral tests related to depression were conducted to investigate the antidepressant impact of ODEXs (Figure 1F). In the SPT, the CUMS group exhibited a decrease in sucrose preference, and the administration of ODEXs reversed this reduction (Figure 1G). In the FST, the CUMS group showed a notable increase in total immobility time, but this was significantly mitigated by ODEX treatment (Figure 1H). In the OFT, CUMS reduced the center time but had no effect on the total distance for different groups (Figure 1I,J). These results suggested that ODEXs effectively alleviated the behavioral deficits observed in CUMS mice.

### 3.3 ODEXs restore neurogenesis and synaptic plasticity in CUMS mice

**FIGURE 2:**
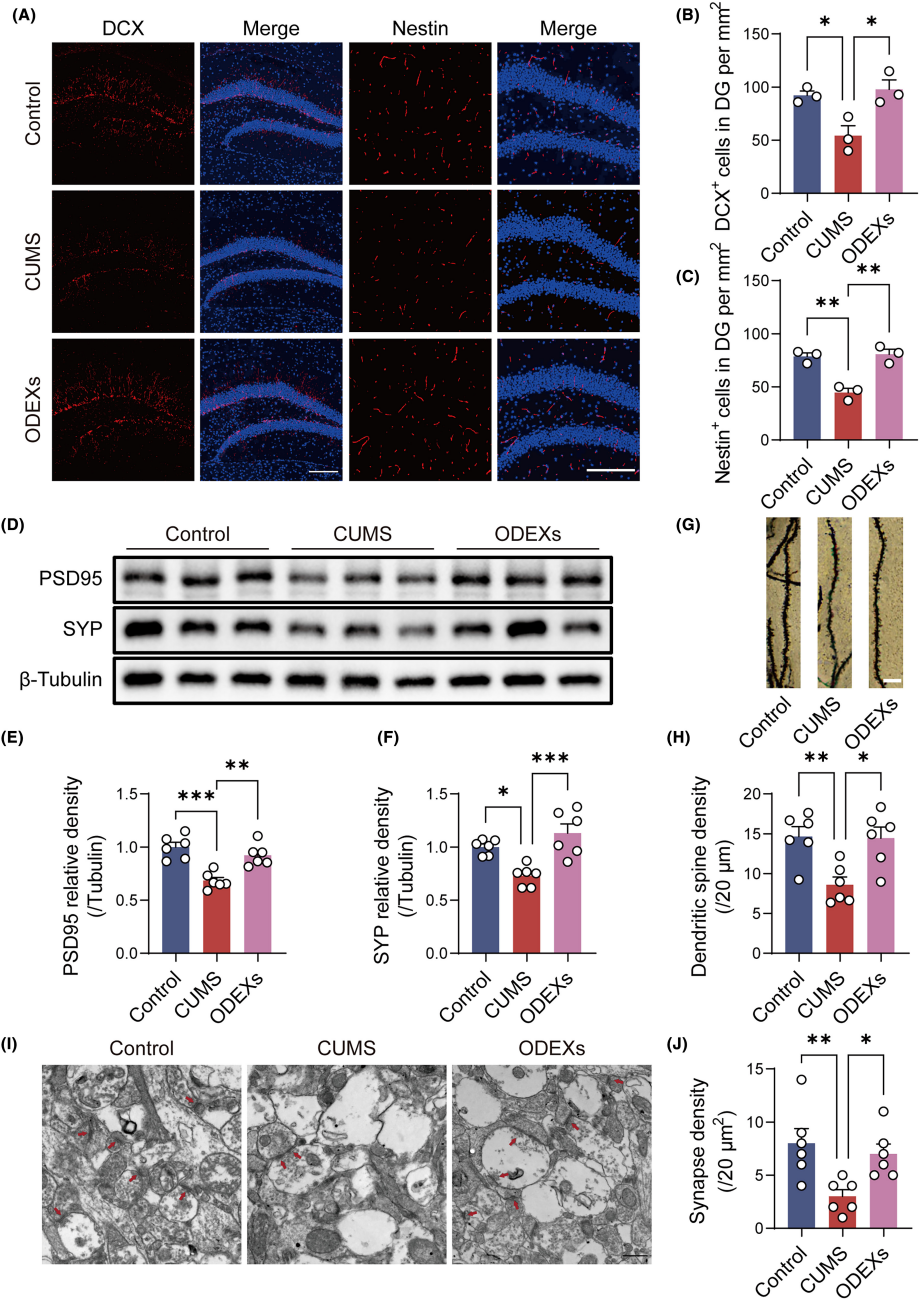
ODEXs promote neurogenesis and synaptic plasticity in the hippocampus of CUMS mice. (A) Immunofluorescence images of DCX^+^ and Nestin^+^ cells in the DG. Scale bar, 200 μm. (B, C) Quantification of the number of DCX^+^ and Nestin^+^ cells in the DG per mm^2^ (*n* = 3). (D) Western blotting analysis shows that ODEX treatment reversed reductions in SYP and PSD95 in CUMS mice. Full‐unedited gels are presented in Figure S2. (E, F) Quantification of the PSD95/tubulin and SYP/tubulin ratios (*n* = 6). (G) Golgi‐Cox‐stained images of dendritic spines. Scale bar, 5 μm. (H) Quantification of dendritic spine density (*n* = 6). (I) TEM images of the hippocampal synapses. Scale bar, 500 nm. (J) Quantification of synapse density (*n* = 6). Data are expressed as mean ± SEM. *p < 0.05, **p < 0.01, ***p < 0.001 by one‐way ANOVA with Tukey's multiple‐comparisons test.

Immunofluorescence analysis demonstrated that the number of DCX^+^ and Nestin^+^ cells decreased in CUMS mice, and ODEX treatment reversed this reduction (Figure 2A–C). Western blotting results revealed reductions in PSD95 and SYP expression in CUMS mice, while ODEX treatment restored the expression of these synaptic proteins to control levels (Figure 2D–F). Golgi‐Cox staining showed that ODEX treatment significantly ameliorated the reduction in dendritic spine density in CUMS mice (Figure 2G,H). TEM revealed a reduction in the number of hippocampal synapses in CUMS mice, and synapse density was rescued after ODEX treatment (Figure 2I,J). Overall, these findings indicated that ODEXs can restore neuroplasticity impairments in CUMS mice.

### 3.4 SIRT2 is abundant in ODEXs and plays a key role in the antidepressant‐like effects of ODEXs

**FIGURE 3:**
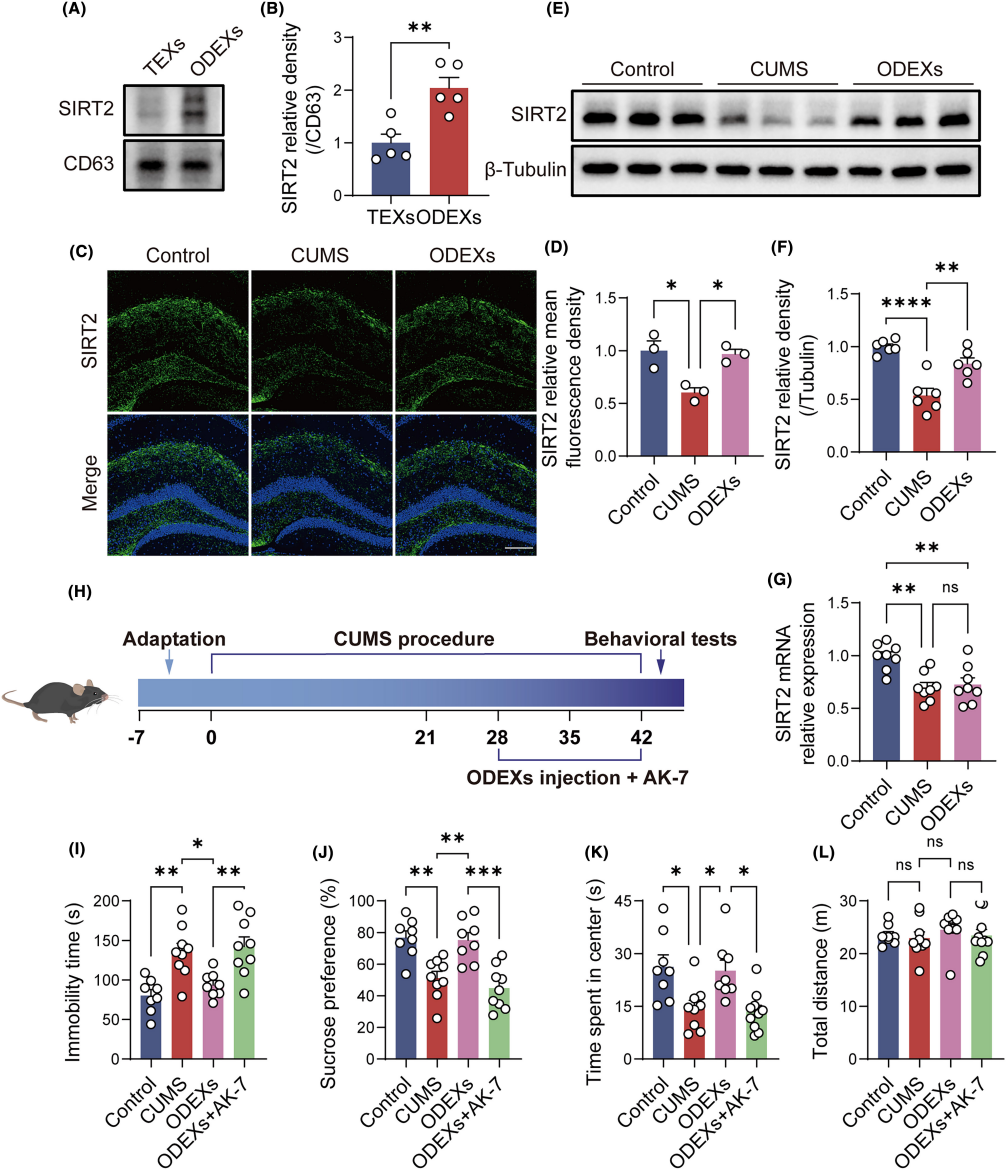
SIRT2 is the key mediator of the antidepressant‐like effects of ODEXs. (A) Western blotting analysis of SIRT2 and CD63 in TEXs and ODEXs. Full‐unedited gels are presented in Figure S3. (B) Quantification of the SIRT2/CD63 ratio (*n* = 5). (C) Immunofluorescence images of SIRT2 in the hippocampus. Scale bar, 200 μm. (D) Quantification of the relative mean fluorescence intensity of SIRT2 (*n* = 3). (E) Western blotting analysis of SIRT2 in the hippocampus. Full‐unedited gels are presented in Figure S4. (F) Quantification of the SIRT2/tubulin ratio (*n* = 6). (G) RT‐PCR analysis of SIRT2 in the hippocampus (*n* = 8). (H) Schematic of the animal experiments. (I–L) Effects of AK‐7 on the antidepressant‐like effects of ODEXs in the SPT, FST, and OFT (*n* = 8–10). Data are expressed as mean ± SEM. ns, not significant, *p < 0.05, **p < 0.01, ***p < 0.001, and ****p < 0.0001 by unpaired Student's *t*‐test and one‐way ANOVA with Tukey's multiple‐comparisons test.

Oligodendrocyte‐derived exosomes were enriched for SIRT2, as identified by western blotting (Figure 3A,B). Moreover, western blotting and immunofluorescence staining indicated that ODEX treatment restored decreases in hippocampal SIRT2 in CUMS mice (Figure 3C–F). Notably, RT‐PCR revealed that ODEX treatment had no significant effect on hippocampal SIRT2 mRNA levels in CUMS mice (Figure 3G).

Behavioral tests were repeated in the presence of AK‐7, a selective inhibitor of SIRT2 (Figure 3H). SIRT2 blockade eliminated the antidepressant‐like impact of ODEXs in the SPT, FST, and OFT (Figure 3I–L). Taken together, SIRT2 appears to be essential for the antidepressant‐like effects of ODEXs.

### 3.5 ODEXs promote neurogenesis and synaptic plasticity through SIRT2‐mediated AKT/GSK‐3β signaling in CUMS mice

**FIGURE 4:**
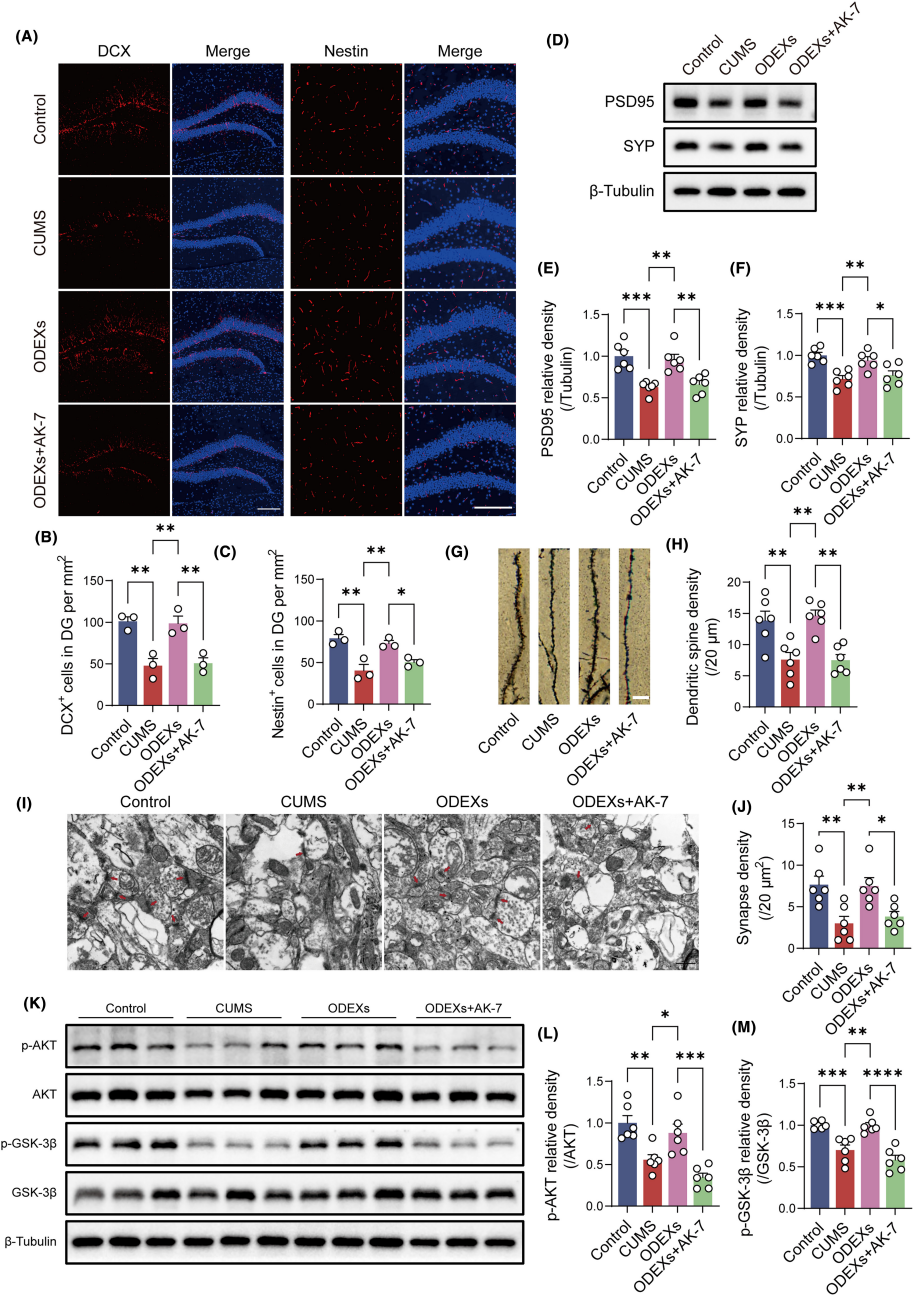
ODEXs restore neuroplasticity impairments via SIRT2‐mediated AKT/GSK‐3β signaling. (A) Immunofluorescence images of DCX^+^ and Nestin^+^ cells in the DG. Scale bar, 200 μm. (B, C) Quantification of the number of DCX^+^ and Nestin^+^ cells in the DG per mm^2^ (*n* = 3). (D) Western blotting analysis showing SIRT2 inhibition prevents increases in SYP and PSD95 in CUMS mice after ODEX treatment. Full‐unedited gels are presented in Figure S5. (E, F) Quantification of the PSD95/tubulin and SYP/tubulin ratios (*n* = 6). (G) Golgi‐Cox‐stained images of dendritic spines. Scale bar, 5 μm. (H) Quantification of dendritic spine density (*n* = 6). (I) TEM images of the hippocampal synapses. Scale bar, 500 nm. (J) Quantification of synapse density (*n* = 6). (K) Western blotting analysis of p‐AKT, AKT, p‐GSK‐3β, and GSK‐3β among different groups. Full‐unedited gels are presented in Figure S6. (L, M) Quantification of the p‐AKT/AKT and p‐GSK‐3β/GSK‐3β ratios (*n* = 6). Data are expressed as mean ± SEM. *p < 0.05, **p < 0.01, ***p < 0.001, and ****p < 0.0001 by one‐way ANOVA with Tukey's multiple‐comparisons test.

To confirm SIRT2's function in neurogenesis and synaptic plasticity via ODEXs, we repeated morphometric and molecular analyses in the presence of AK‐7, a selective cell‐ and brain‐permeable SIRT2 inhibitor widely used via intraperitoneal injection in preclinical studies of central nervous system diseases.^36^, ^37^, ^38^, ^39^, ^40^, ^41^ Immunofluorescence analysis indicated SIRT2 inhibition abolished the ability of ODEXs to increase newly developed cells in CUMS mice (Figure 4A–C). SIRT2 inhibition also eliminated the beneficial impact of ODEXs on synaptic plasticity in CUMS mice, as verified by western blotting, Golgi‐Cox staining, and TEM (Figure 4D–J).

Next, we investigated the underlying mechanism modulating neuroplasticity. The AKT/GSK‐3β pathway is essential for regulating neuroplasticity, and it has been reported that SIRT2 can directly bind to AKT to regulate its activation.^42^, ^43^ Western blotting revealed that p‐AKT and p‐GSK‐3β expression increased in CUMS mice after ODEX treatment, and AK‐7 reduced the expression of these phosphorylated proteins and inhibited AKT/GSK‐3β signaling (Figure 4K–M). ODEXs appear to promote neuroplasticity through SIRT2‐mediated AKT/GSK‐3β signaling in CUMS mice.

### 3.6 Increased SIRT2 levels in NSPCs are associated with enhanced neurogenesis and synaptic protein expression

**FIGURE 5:**
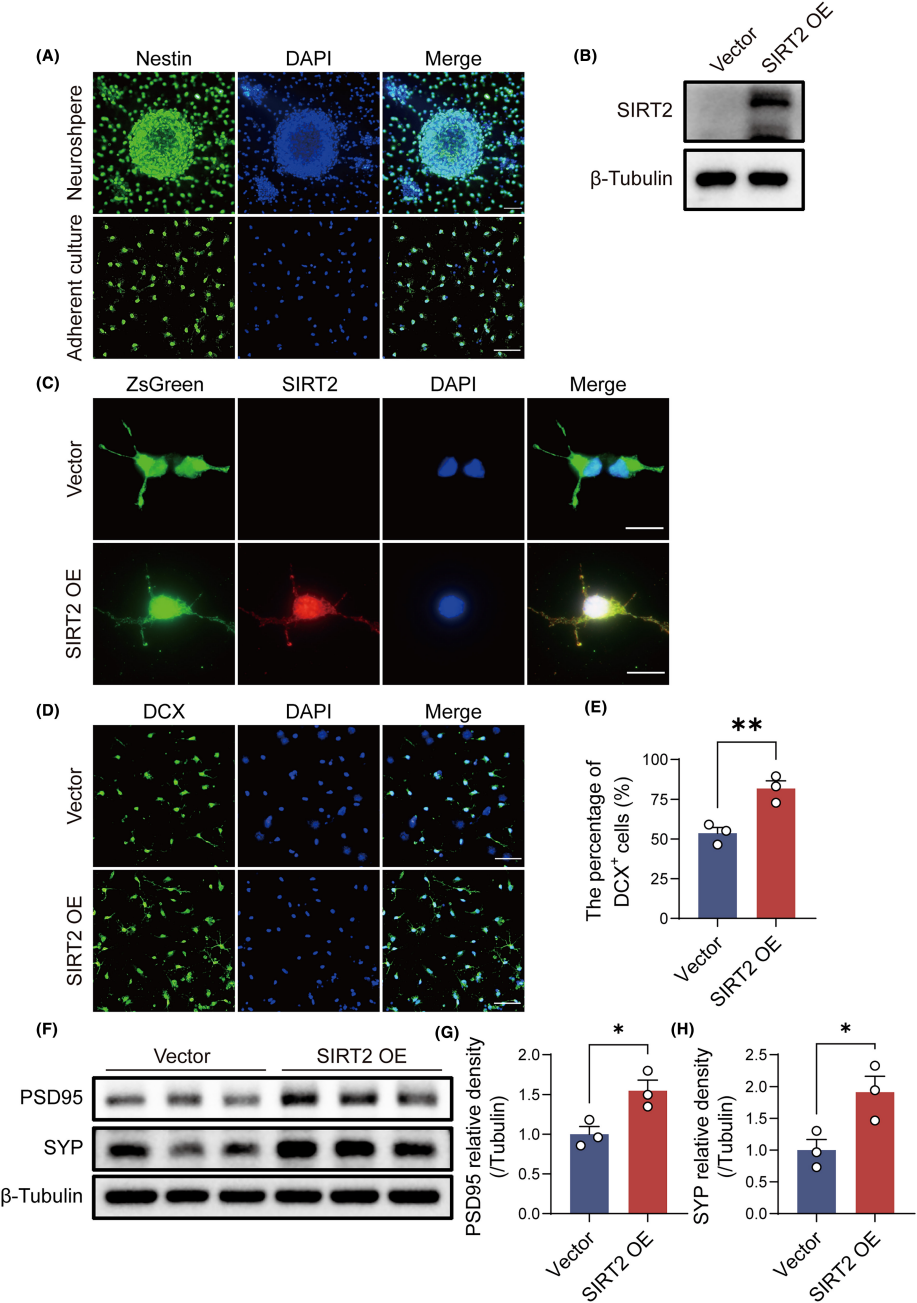
Elevated SIRT2 expression in NSPCs promotes neurogenesis and synapse formation. (A) Representative images of Nestin in NPSCs. Scale bars, 50 μm. (B) Western blotting analysis of SIRT2. Full‐unedited gels are presented in Figure S7. (C) Representative images of SIRT2 in NPSCs. Scale bars, 10 μm. (D) Immunofluorescence images of DCX^+^ cells in NPSCs transfected with the vector or SIRT2 lentivirus. Scale bar, 50 μm. (E) Quantification of the percentages of DCX^+^ cells per DAPI^+^ cells. (F) Western blotting analysis of PSD95 and SYP. Full‐unedited gels are presented in Figure S8. (G, H) Quantification of the PSD95/tubulin and SYP/tubulin ratios. Data are expressed as mean ± SEM (*n* = 3). ns, not significant, *p < 0.05 and **p < 0.01 by unpaired Student's *t*‐test.

To investigate the potential effect of SIRT2 on neurogenesis and synapse formation, we infected NSPCs with recombinant lentivirus vector VP032‐harboring *Sirt2* or negative controls. Primary NSPCs were confirmed by intense Nestin expression (Figure 5A). Western blotting and immunofluorescence staining demonstrated that SIRT2 was undetectable in NSPCs, while transfection with SIRT2 lentivirus successfully induced intense SIRT2 expression (Figure 5B,C). Moreover, the number of DCX^+^ cells increased in NSPCs‐overexpressing SIRT2 (Figure 5D,E). After 14 days of differentiation, western blotting revealed that SYP and PSD95 expression significantly increased in NSPCs‐overexpressing SIRT2 (Figure 5F–H). These results support that elevated SIRT2 in NSPCs is sufficient to enhance neurogenesis and synaptic protein expression.

### 3.7 ODEXs promote neurogenesis and synapse formation in NSPCs, while SIRT2 inhibition reverses these effects through AKT/GSK‐3β signaling

**FIGURE 6:**
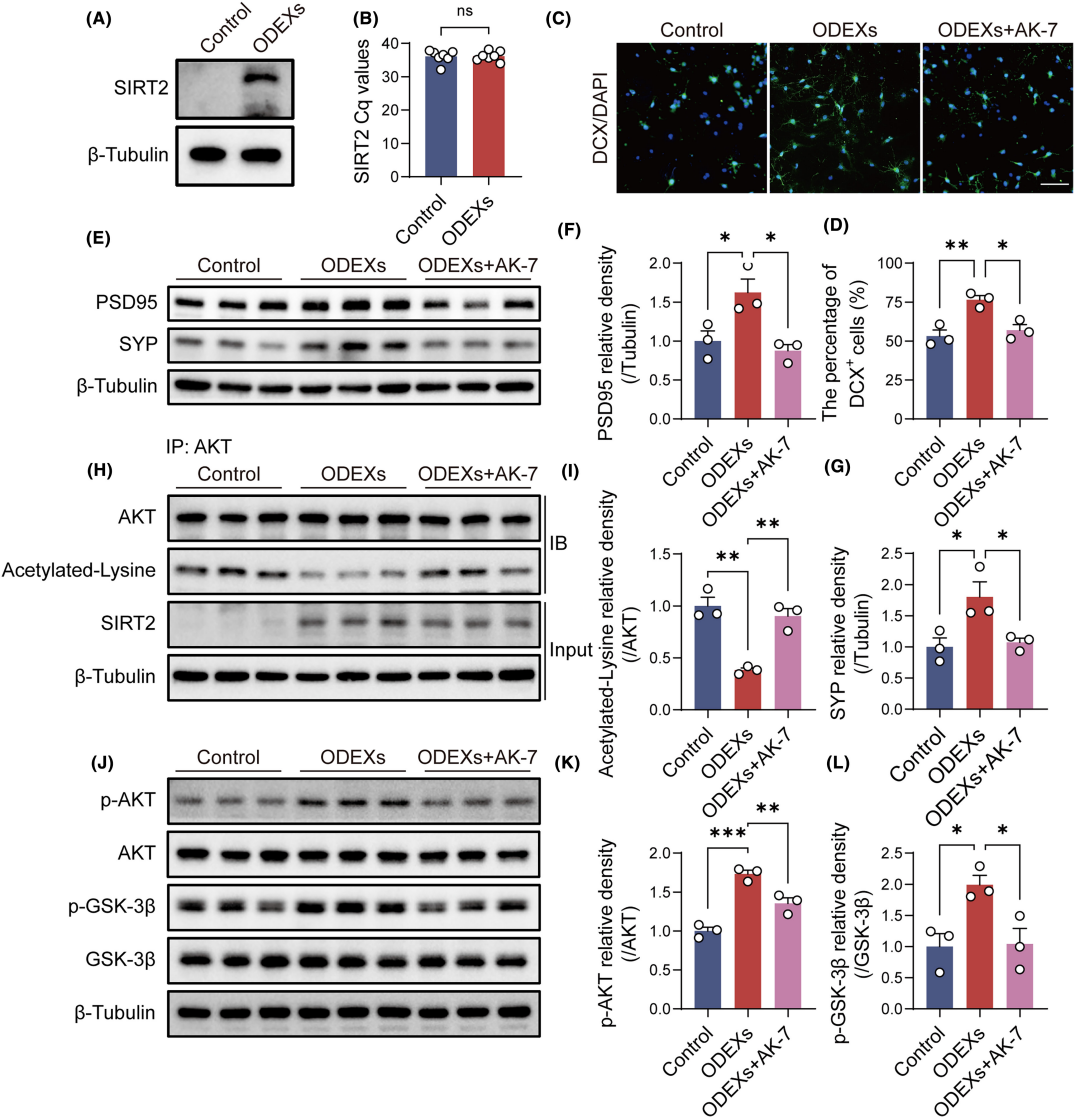
ODEXs enhance neurogenesis and synaptic protein expression, and SIRT2 inhibition prevents these effects via SIRT2‐mediated AKT/GSK‐3β signaling in NSPCs. (A) Western blotting analysis of SIRT2. Full‐unedited gels are presented in Figure S9. (B) RT‐PCR analysis of SIRT2. (*n* = 7) (C) Immunofluorescence images of DCX^+^ cells in the different groups. Scale bar, 50 μm. (D) Quantification of the percentages of DCX^+^ cells per DAPI^+^ cells. (E) Western blotting analysis of PSD95 and SYP. Full‐unedited gels are presented in Figure S10. (F, G) Quantification of the PSD95/tubulin and SYP/tubulin ratios. (H) Immunoprecipitation analysis of acetylated AKT and western blotting analysis of SIRT2. Full‐unedited gels are presented in Figure S11. (I) Quantification of the acetylated‐lysine/AKT ratio. (J) Western blotting analysis of p‐AKT, AKT, p‐GSK‐3β, and GSK‐3β. Full‐unedited gels are presented in Figure S12. (K, L) Quantification of the p‐AKT/AKT and p‐GSK‐3β/GSK‐3β ratios. Data are expressed as mean ± SEM (*n* = 3). ns, Not significant, *p < 0.05, **p < 0.01, ***p < 0.001 by one‐way ANOVA with Tukey's multiple‐comparisons test.

To confirm the transcellular delivery of SIRT2 within ODEXs to neurons, the effects of ODEXs were investigated in vitro in cultured NSPCs. Western blotting demonstrated that SIRT2 was undetectable in NSPCs, while ODEX treatment increased SIRT2 protein levels (Figure 6A). RT‐PCR analysis showed that SIRT2 mRNA levels in cells after ODEX treatment were still undetectable (Figure 6B). These results indicated that the expression of SIRT2 is directly mediated by the delivery of SIRT2 protein via ODEXs.

To confirm the in vivo results, we incubated NSPCs with ODEXs and AK‐7. Immunofluorescence staining demonstrated an increased number of DCX^+^ cells in NSPCs after ODEX treatment, and AK‐7 reversed this upregulation (Figure 6C,D). ODEX treatment also increased expression of SYP and PSD95 in NSPCs after neural differentiation, while SIRT2 inhibition reduced the expression of these synaptic proteins (Figure 6E–G). Immunoprecipitation assays revealed that ODEX treatment significantly reduced the expression of acetylated AKT, while AK‐7 administration reversed this downregulation (Figure 6H,I). Moreover, p‐AKT and p‐GSK‐3β expression increased in NSPCs after ODEX treatment, while AK‐7 reduced expression of these phosphorylated proteins (Figure 6J–L). These findings were consistent with the in vivo experiments and demonstrated that ODEXs regulate neuroplasticity through SIRT2‐mediated AKT/GSK‐3β signaling.

## 4 DISCUSSION

Here, we present the first compelling evidence of an antidepressant role for ODEXs, as well as an underlying mechanism, in CUMS mice. Our results show that: (1) ODEXs reverse CUMS‐induced depressive‐like behaviors and hippocampal neuroplasticity; (2) SIRT2 is enriched in ODEXs, and blocking SIRT2 using AK‐7 reverses the antidepressant effects of ODEXs; (3) overexpression of SIRT2 is sufficient to enhance neurogenesis and synaptic protein expression; and (4) ODEXs mediate the delivery of SIRT2 protein to neurons to regulate neuroplasticity through the AKT/GSK‐3β pathway.

Our results add to a growing body of evidence that exosomes can exert antidepressant‐like behaviors. For example, NK cell exosomes containing miR‐207 inhibited the release of pro‐inflammatory factors from astrocytes, improving depressive‐like behaviors in mice after chronic mild stress.^44^ Guo et al.^45^ found that bone marrow mesenchymal stem cell‐derived exosomes alleviated depressive‐like behaviors in depressed rats. Clinical studies on the effects of exosomes from depressed patients on depressive‐like behaviors are conflicting,^46^, ^47^ but one study showed that exosomes from patients with depression are enriched with the sigma‐1 receptor, which can exert antidepressant effects via sigma‐1 receptor delivery.^46^ Conversely, another study found that exosomes from patients with depression inhibit neurogenesis in mice, inducing depressive‐like behaviors.^47^

As depression progresses, impairments in neuroplasticity can be detected in multiple brain regions, especially the hippocampus.^48^ The hippocampus, recognized as a central hub for adult neurogenesis and mood regulation, is strongly linked to the onset of depression and the efficacy of antidepressant interventions.^5^ Consistent with previous studies, we effectively established the CUMS‐induced mouse model of depression.^49^ These behavioral changes in CUMS mice were associated with a decrease in newly developed hippocampal cells and synapse impairments.^48^

Exosomes can facilitate intercellular communication between various cell types, and they play a significant role in neuroplasticity in various pathophysiological conditions, including depression.^13^, ^50^, ^51^ Our study demonstrated that tail vein injection of ODEXs ameliorated depressive‐like behaviors in CUMS mice. Furthermore, ODEX treatment counteracted the neurogenesis impairment induced by chronic stress, resulting in an augmented population of DCX^+^ and Nestin^+^ cells in the DG, indicative of enhanced neural stem cell (NSC) proliferation and neuronal differentiation.^13^ Cells derived from the NSC neuron lineage are known to be involved in emotion, cognition, and behavior.^52^, ^53^ Moreover, we found that ODEX treatment restored synaptic dysfunction, as evidenced by increased expression of PSD95 and SYP, enhanced dendritic spine density, and an augmented number of hippocampal synapses. Overall, these findings indicate promise for ODEXs as a treatment for depression. Previous studies have shown that neurons can internalize ODEXs along with their protein cargoes to increase neuron viability and stress resilience.^19^, ^20^ Proteomic analysis has also revealed enrichment of myelin components and proteins related to metabolism, signaling, and cellular stress response within these exosomes.^54^ Therefore, the protein cargo within ODEXs is likely to be essential for their biological effects.

Previous proteomic and molecular findings have confirmed that SIRT2 is enriched in ODEXs and that its delivery from oligodendrocytes to neurons via exosomes is vital in the CNS.^16^, ^54^ SIRT2 is highly expressed in various brain areas including the cortex, frontal lobe, hippocampus, and striatum.^55^ It can regulate various hippocampal functions including cell proliferation, NSC differentiation, synaptic plasticity, and memory formation.^56^, ^57^, ^58^ The substrates and functions of SIRT2 continue to be discovered, but its role in MDD remains controversial.^22^, ^59^ Guclu et al.^60^ found that inhibiting SIRT2 upregulated the expression of genes and proteins associated with neuroplasticity and neurotrophic factors, exerting an antidepressant‐like effect in mice. However, it has also been shown that chronic stress downregulates hippocampal SIRT2 expression, while its overexpression reversed depressive‐like behaviors induced by chronic stress and promoted neurogenesis.^26^ These opposite roles of SIRT2 suggest a context‐dependent pleiotropic function. There have been no previous studies on oligodendrocyte‐derived exosomal SIRT2 for depression. Our findings suggest that SIRT2 is indeed a critical component of ODEXs and can ameliorate depressive‐like behaviors in CUMS mice. Additionally, treating ODEXs with AK‐7 led to a reduction in pro‐neurogenic effects, as evidenced by a decrease in DCX^+^ and Nestin^+^ cells. Similarly, Zhao et al.^61^ revealed that SIRT2 participates in regulating the proliferation and differentiation of NSCs in ischemic stroke. With respect to the role of SIRT2 in synaptic plasticity, SIRT2 knockout mice display abnormal synaptic plasticity accompanied by learning and memory impairments.^57^ Yang et al.^41^ also found that SIRT2 can regulate synapse formation in white matter injury models. Furthermore, many studies have confirmed the critical function of exosomes in improving synaptic damage.^62^, ^63^ Our research extends this understanding by revealing that ODEXs can enhance synaptic plasticity, and SIRT2 inhibition can attenuate these beneficial effects both in vivo and in vitro. This further validates the mechanistic role of ODEXs in improving depression.

Many studies have highlighted the important role played by AKT/GSK‐3β signaling in regulating neuroplasticity and its role in the pathogenesis of depression.^64^ GSK‐3β, a downstream target of AKT, is an important protein kinase that is highly expressed in the CNS and that participates in numerous cellular processes.^65^ AKT activation can phosphorylate GSK‐3β at serine 9, inhibiting its activity and enhancing neuronal function and synaptic plasticity.^66^, ^67^ Conversely, decreased AKT phosphorylation is associated with GSK‐3β activation and impaired neural development.^68^ Moreover, SIRT2 can directly bind to AKT to regulate its acetylation and phosphorylation levels in different cell types.^42^, ^43^, ^69^ In this study, we observed that the ODEX‐mediated upregulation of SIRT2 reduced the acetylation and increased phosphorylation of AKT, followed by suppression of GSK‐3β activity. Notably, the administration of AK‐7, which inhibits SIRT2, effectively reversed the activation of this signaling pathway. Hence, these findings offer a credible explanation for the neuroplasticity‐related functions of ODEXs through the SIRT2‐mediated AKT/GSK‐3β pathway.

This study has several limitations. First, our research primarily focused on the impact of ODEXs on hippocampal neurogenesis and synaptic plasticity in depression, without exploring their potential effects on myelination, oxidative stress, and neuroinflammation. Second, we did not investigate the effects of ODEXs in normal mice, and investigating the impact of varying concentrations of exosomes on serum ODEX levels in mice would be worth investigating. Comparing their physiological and pathological roles in future research will be of significant value. Third, our study exclusively utilized AK‐7 to inhibit SIRT2, which, although established as a selective cell‐ and brain‐permeable SIRT2 inhibitor, could be supported with further studies of SIRT2 knockout in the model system. We did not directly visualize delivery process of SIRT2 within ODEXs in vivo, and further validation is required using SIRT2‐deficient exosomes and anti‐SIRT2 immunogold electron microscopy. Fourth, although we used biochemical readouts of synaptic plasticity, long‐term potentiation, and long‐term depression metrics would be valuable to support these findings. Finally, we only studied male mice to overcome the potential impact of female sex hormones on mood, cognition, and behavior. Further experiments are needed to assess sex‐related differences in ODEX function.

## 5 CONCLUSION

In summary, here we establish that ODEXs alleviate depressive‐like behaviors and improve hippocampal neurogenesis and synaptic plasticity following CUMS in mice. These beneficial effects of ODEXs appear to be due to delivery of SIRT2 and activation of AKT/GSK‐3β signaling. These findings identify a new mechanism for ODEXs in the treatment of depression and provide a promising therapeutic approach for depressed patients.

## AUTHOR CONTRIBUTIONS

Honghan Zhang, Di Li, and Zhongchun Liu conceptualized and designed the study. Honghan Zhang carried out the experiments, performed data analysis, and wrote the manuscript. Chao Wang, Ningyuan Li, Yuqi Feng, and Siqi Sun completed some experiments. Xinhua Song, Ruiling Li, and Hao Duan recorded and analyzed the behavioral data. Zhongchun Liu, Xinhui Xie, and Xu Shuxian revised the manuscript. All authors read and approved the final manuscript.

## FUNDING INFORMATION

This work was supported by grants from the National Natural Science Foundation of China (U21A20364) and the National Key R&D Program of China (2018YFC1314600).

## CONFLICT OF INTEREST STATEMENT

The authors declare no conflicts of interest.

## Supporting information




Figures S1–S12

Tables S1–S4

## Data Availability

The data that support the findings of this study are available from the corresponding author upon reasonable request.
