# Supplementary material for: Oligodendrocyte‐derived exosomes‐containing SIRT2 ameliorates depressive‐like behaviors and restores hippocampal neurogenesis and synaptic plasticity via the AKT/GSK‐3β pathway in depressed mice
Source: CNS Neurosci Ther. 2024 Mar 4;30(3):e14661. doi: 10.1111/cns.14661 (PMC10912796; doi:10.1111/cns.14661)
Supplement: Supplementary file 1 — Figures S1–S12 [file CNS-30-e14661-s001.zip › Supplement File 1-Revision.pdf]

Figure S1

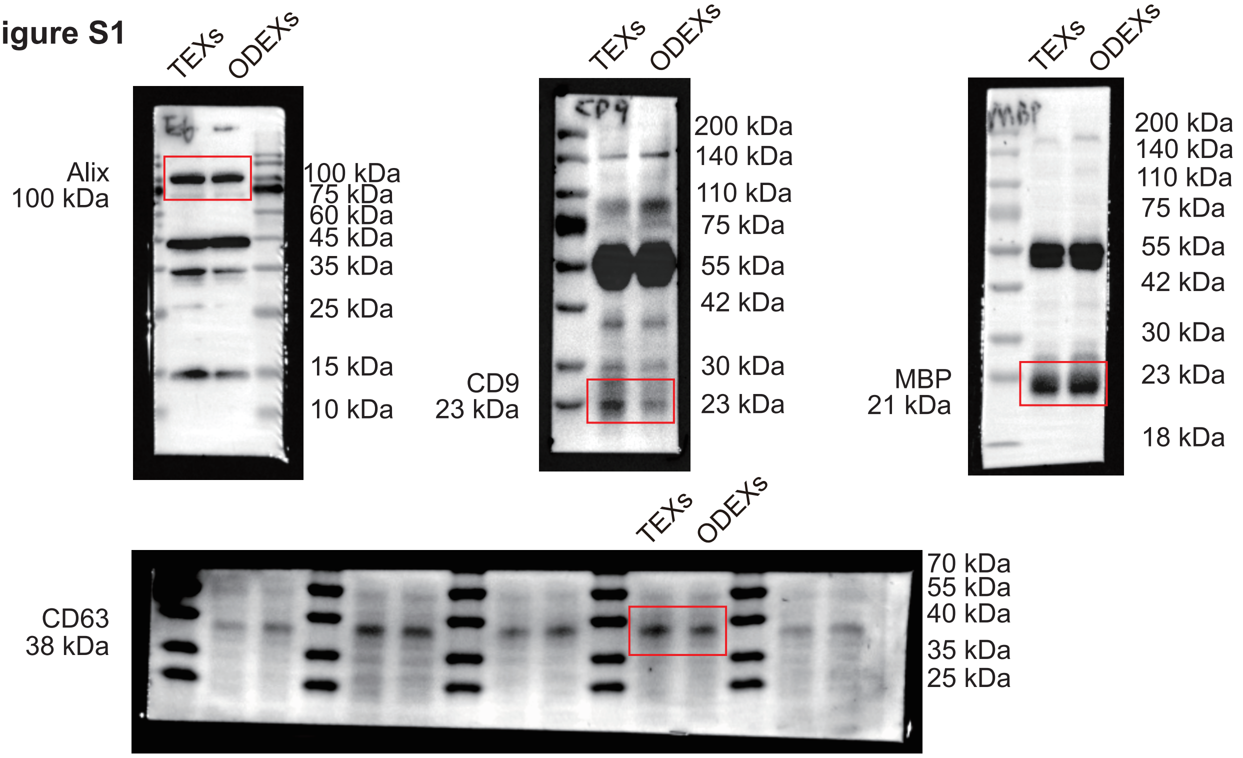

Figure S1. Full unedited gels of Figure 1C.
Western blotting analysis of CD9, CD63, Alix, and MBP in TEXs and ODEXs.

Figure S2

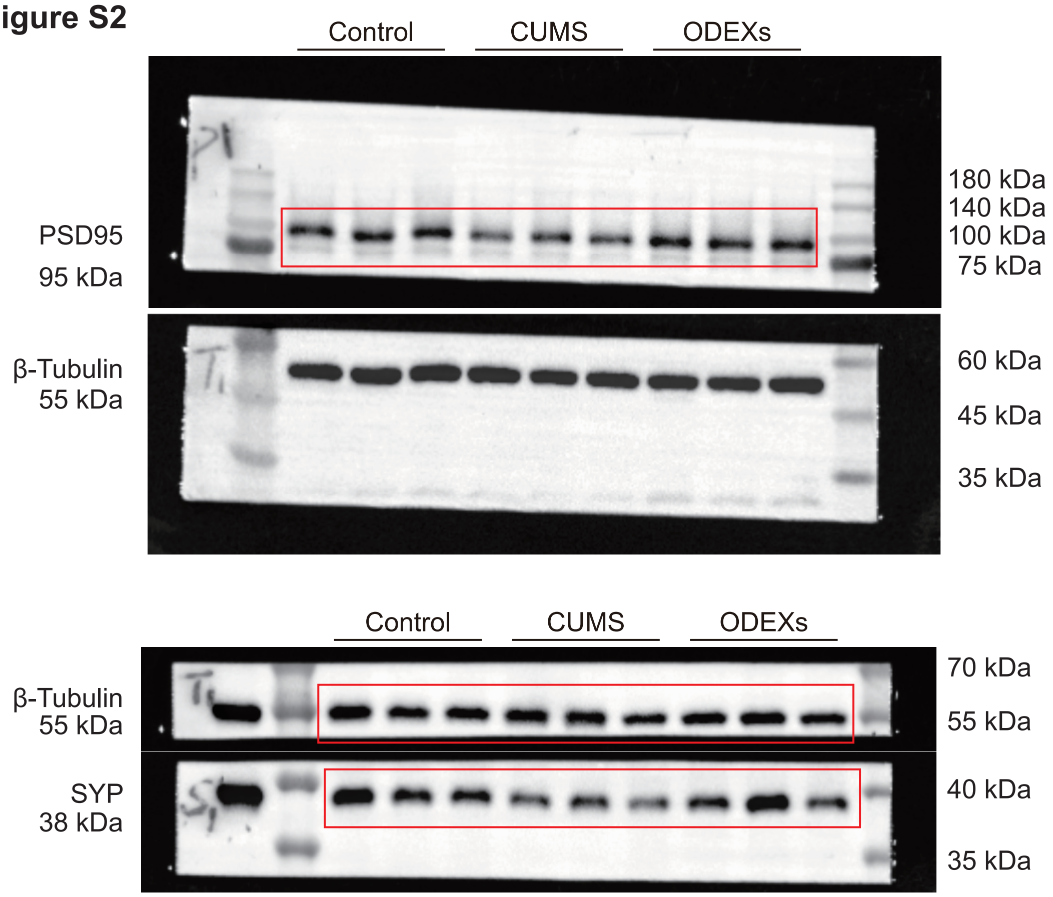

Figure S2. Full unedited gels of Figure 2D.
Western blotting analysis of SYP and PSD95 in Control, CUMS and ODEXs groups of mice.

Figure S3

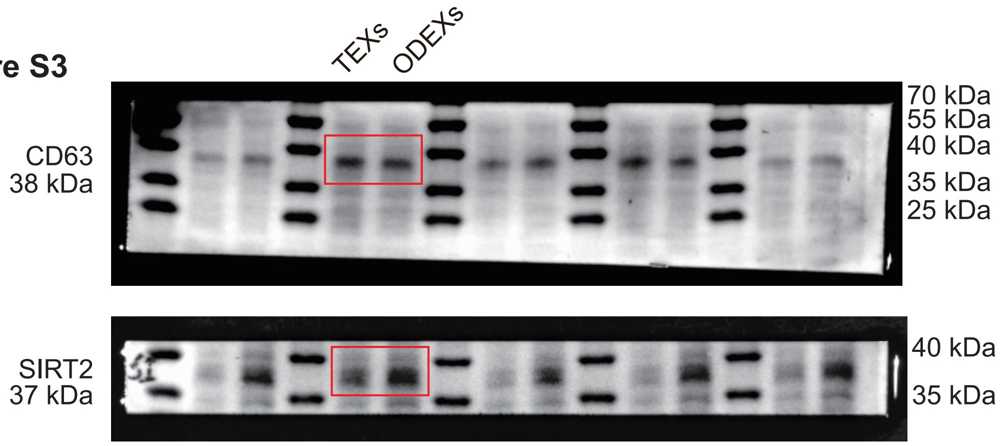

Figure S3. Full unedited gels of Figure 3A.
Western blotting analysis of SIRT2 and CD63 in TEXs and ODEXs.

Figure S4

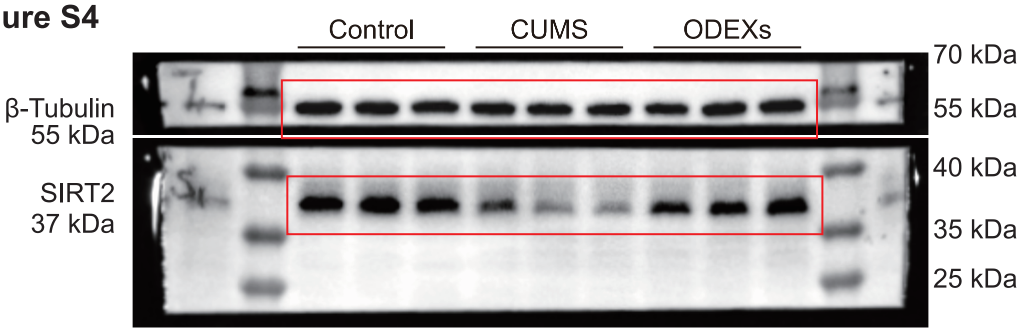

Figure S4. Full unedited gels of Figure 3E.
Western blotting analysis of SIRT2 in Control, CUMS and ODEXs groups of mice.

Figure S5

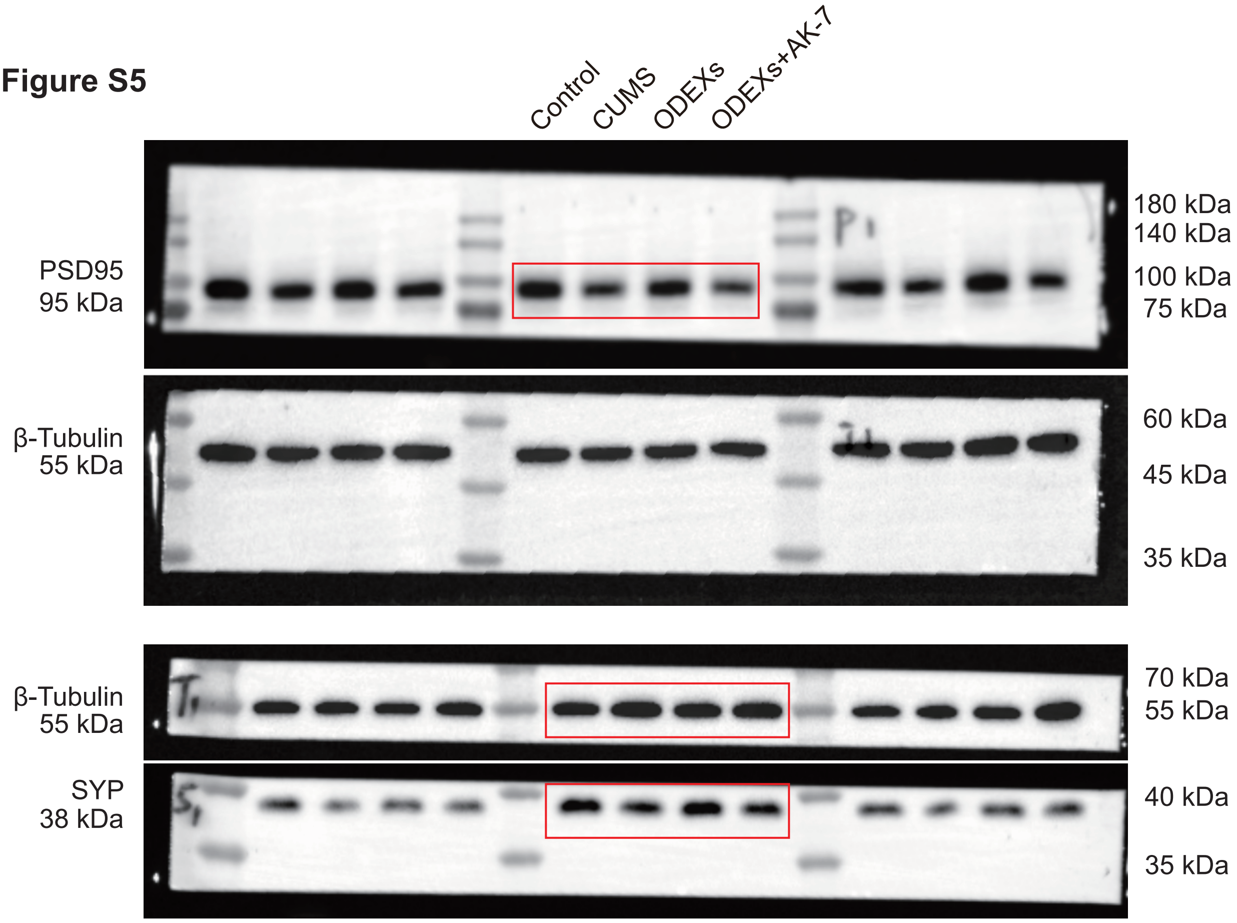

Figure S5. Full unedited gels of Figure 4D.
Western blotting analysis of SYP and PSD95 in Control, CUMS, ODEXs and ODEXs + AK-7 groups of mice.

Figure S6

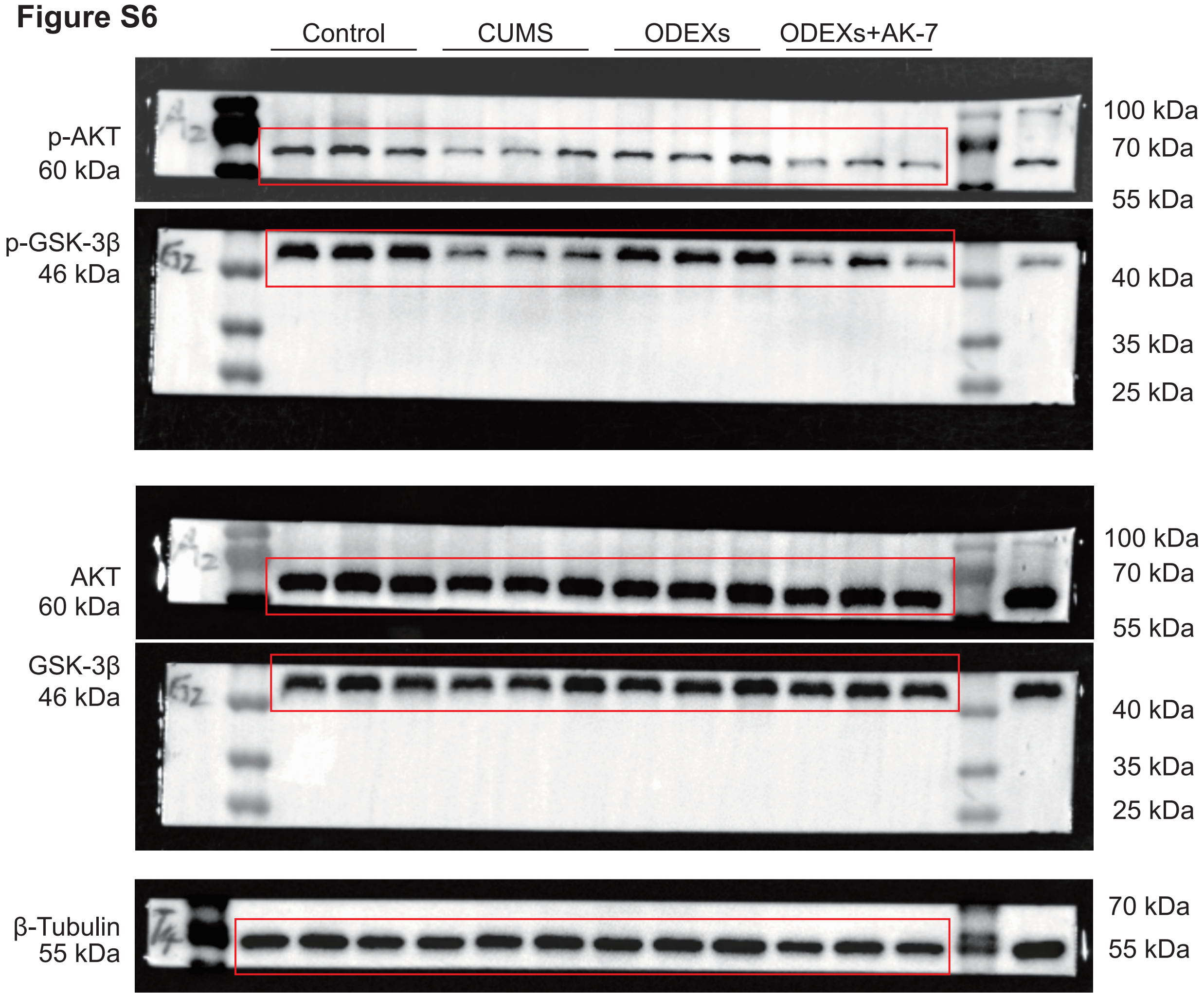

Figure S6. Full unedited gels of Figure 4K.
Western blotting analysis of p-AKT, AKT, p-GSK-3β and GSK-3β in Control, CUMS, ODEXs and ODEXs + AK-7 groups of mice.

Figure S7

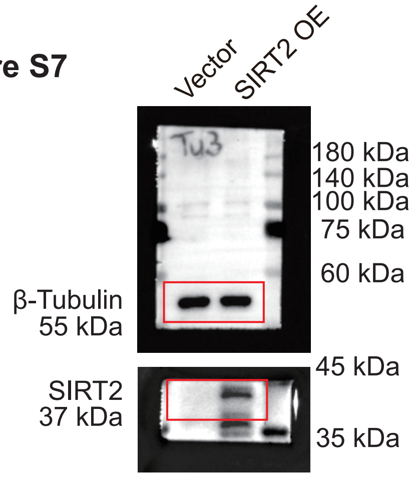

Figure S7. Full unedited gels of Figure 5B.
Western blotting analysis of SIRT2 in vector and SIRT2 lentivirus transfected NSPCs.

Figure S8

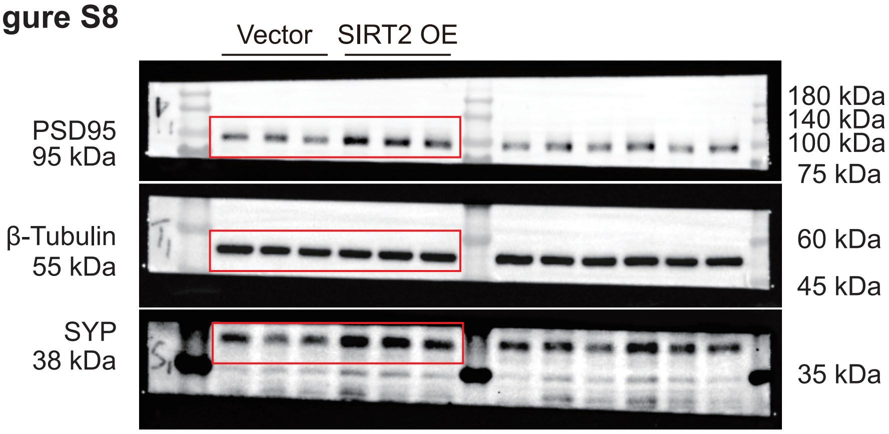

Figure S8. Full unedited gels of Figure 5F.
Western blotting analysis of SYP and PSD95 in vector and SIRT2 lentivirus transfected NSPCs.

Figure S9

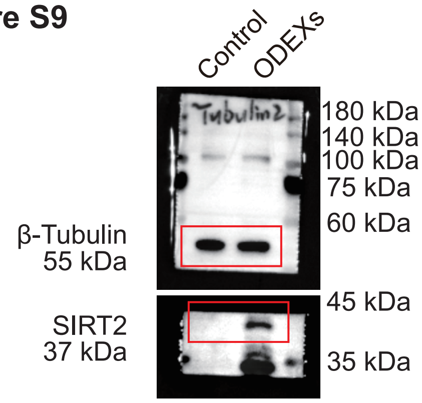

Figure S9. Full unedited gels of Figure 6A.
Western blotting analysis of SIRT2 in Control and ODEXs groups of NSPCs.

Figure S10

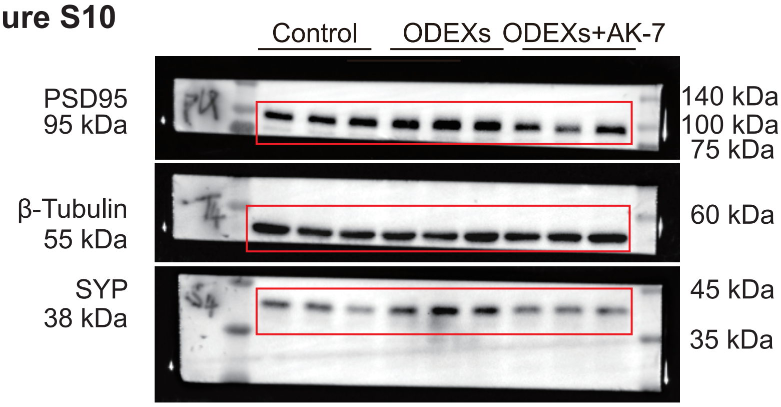

Figure S10. Full unedited gels of Figure 6E.
Western blotting analysis of SYP and PSD95 in Control, ODEXs and ODEXs + AK-7 groups of NSPCs.

Figure S11

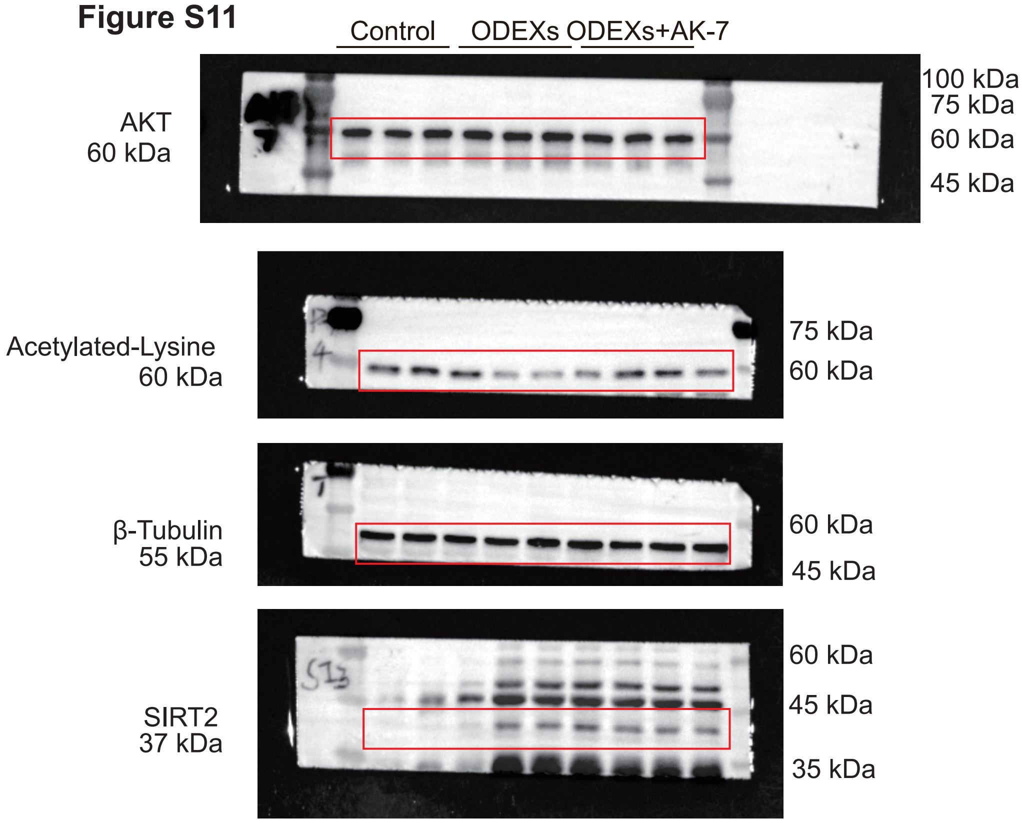

Figure S11. Full unedited gels of Figure 6H.
Immunoprecipitation analysis of acetylated AKT and western blotting analysis of SIRT2 in Control, ODEXs and ODEXs + AK-7 groups of NSPCs.

Figure S12

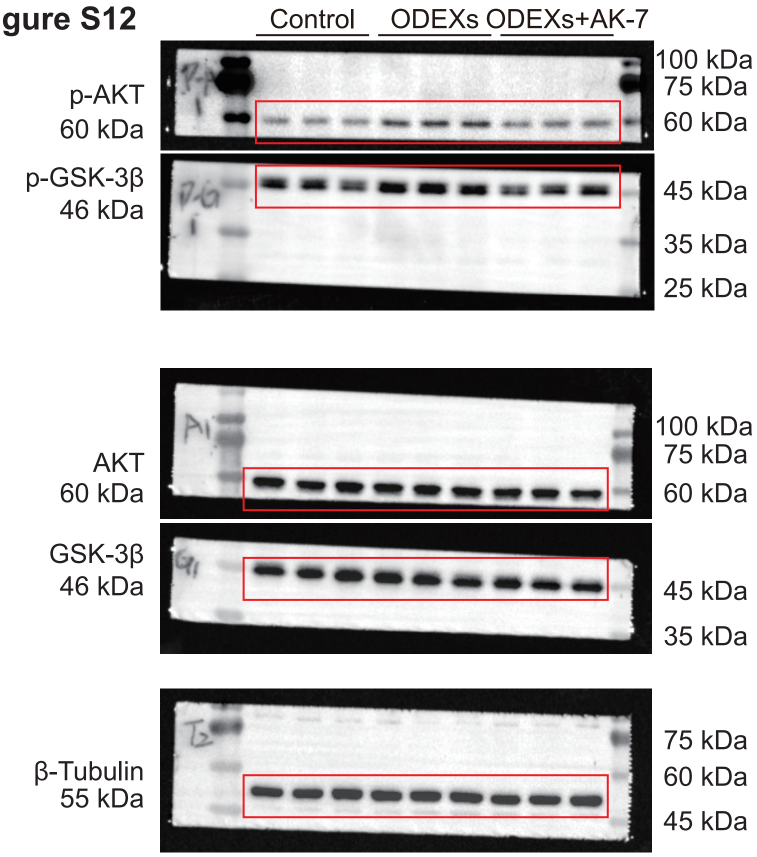

Figure S12. Full unedited gels of Figure 6J.
Western blotting analysis of p-AKT, AKT, p-GSK-3β and GSK-3β in Control, ODEXs and ODEXs + AK-7 groups of NSPCs.
