# Supplementary material for: Oligodendrocyte‐derived exosomes‐containing SIRT2 ameliorates depressive‐like behaviors and restores hippocampal neurogenesis and synaptic plasticity via the AKT/GSK‐3β pathway in depressed mice
Source: CNS Neurosci Ther. 2024 Mar 4;30(3):e14661. doi: 10.1111/cns.14661 (PMC10912796; doi:10.1111/cns.14661)
Supplement: Supplementary file 2 — Tables S1–S4 [file CNS-30-e14661-s002.doc]

**Supplementary Table 1. The specific schedule for CUMS**

| Week | Day | Stressor 1 | Stressor 2 |
| --- | --- | --- | --- |
| 1 | Monday | 24-hour cage tilting at 45° | 6-hour physical restraint |
|  | Tuesday | 24-hour light/dark cycle reversal | 30-min cage shaking at 120 rpm |
|  | Wednesday | 24-hour solid cage | 5-min swimming in 4°C water |
|  | Thursday | 24-hour water deprivation | 10-min tail pinching |
|  | Friday | 24-hour damp bedding | 6-hour physical restraint |
|  | Saturday | 24-hour light/dark cycle reversal | 24-hour cage tilting at 45° |
|  | Sunday | 24-hour food deprivation | 10-min tail pinching |
| 2 | Monday | 24-hour solid cage | 24-hour water deprivation |
|  | Tuesday | 24-hour cage tilting at 45° | 6-hour physical restraint |
|  | Wednesday | 24-hour light/dark cycle reversal | 30-min cage shaking at 120 rpm |
|  | Thursday | 24-hour food deprivation | 10-min tail pinching |
|  | Friday | 24-hour damp bedding | 5-min swimming in 4°C water |
|  | Saturday | 24-hour water deprivation | 6-hour physical restraint |
|  | Sunday | 24-hour food deprivation | 24-hour cage tilting at 45° |
| 3 | Monday | 24-hour solid cage | 10-min tail pinching |
|  | Tuesday | 24-hour light/dark cycle reversal | 30-min cage shaking at 120 rpm |
|  | Wednesday | 24-hour damp bedding | 6-hour physical restraint |
|  | Thursday | 24-hour food deprivation | 5-min swimming in 4°C water |
|  | Friday | 24-hour cage tilting at 45° | 10-min tail pinching |
|  | Saturday | 24-hour water deprivation | 24-hour light/dark cycle reversal |
|  | Sunday | 24-hour damp bedding | 6-hour physical restraint |
| 4 | Monday | 24-hour solid cage | 30-min cage shaking at 120 rpm |
|  | Tuesday | 24-hour cage tilting at 45° | 5-min swimming in 4°C water |
|  | Wednesday | 24-hour water deprivation | 10-min tail pinching |
|  | Thursday | 24-hour food deprivation | 6-hour physical restraint |
|  | Friday | 24-hour light/dark cycle reversal | 30-min cage shaking at 120 rpm |
|  | Saturday | 24-hour damp bedding | 10-min tail pinching |
|  | Sunday | 24-hour cage tilting at 45° | 24-hour water deprivation |
| 5 | Monday | 24-hour food deprivation | 6-hour physical restraint |
|  | Tuesday | 24-hour solid cage | 5-min swimming in 4°C water |
|  | Wednesday | 24-hour water deprivation | 30-min cage shaking at 120 rpm |
|  | Thursday | 24-hour damp bedding | 10-min tail pinching |
|  | Friday | 24-hour light/dark cycle reversal | 24-hour solid cage |
|  | Saturday | 24-hour cage tilting at 45° | 5-min swimming in 4°C water |
|  | Sunday | 24-hour water deprivation | 6-hour physical restraint |
| 6 | Monday | 24-hour food deprivation | 24-hour light/dark cycle reversal |
|  | Tuesday | 24-hour damp bedding | 10-min tail pinching |
|  | Wednesday | 24-hour water deprivation | 30-min cage shaking at 120 rpm |
|  | Thursday | 24-hour solid cage | 5-min swimming in 4°C water |
|  | Friday | 24-hour cage tilting at 45° | 6-hour physical restraint |
|  | Saturday | 24-hour light/dark cycle reversal | 24-hour damp bedding |
|  | Sunday | 24-hour food deprivation | 10-min tail pinching |

**Supplementary Table 2. Details of Antibody and dilution rate for western blotting**

| Antibody | Manufacturer | Product numbers | Dilution Rate |
| --- | --- | --- | --- |
| Rabbit anti-CD9 | Abcam | ab236630 | 1:1000 |
| Rabbit anti-CD63 | Abcam | ab134045 | 1:1000 |
| Mouse anti-Alix | Proteintech | 67715-1-Ig | 1:1000 |
| Rat anti-MBP | Abcam | ab7349 | 1:1000 |
| Rabbit anti-SIRT2 | Cell Signaling Technology | 12650S | 1:2000 |
| Rabbit anti-DCX | Proteintech | 13925-1-AP | 1:1000 |
| Rabbit anti-SYP | Cell Signaling Technology | 5461T | 1:2000 |
| Rabbit anti-PSD95 | Abclonal | A7889 | 1:2000 |
| Rabbit anti-p-AKT | Cell Signaling Technology | 4060T | 1:2000 |
| Rabbit anti-AKT | Cell Signaling Technology | 9272S | 1:2000 |
| Rabbit anti-p-GSK-3β | Cell Signaling Technology | 5558T | 1:2000 |
| Rabbit anti-GSK-3β | Cell Signaling Technology | 12456T | 1:2000 |
| Mouse anti-Acetyl-Lysine | Abclonal | A1525 | 1:1000 |
| Rabbit anti-β-Tubulin | Proteintech | 10094-1-AP | 1:5000 |
| Goat anti-rabbit IgG-HRP | Biosharp | BL003A | 1:5000 |
| Goat anti-mouse IgG-HRP | Biosharp | BL001A | 1:5000 |
| Goat anti-rat IgG-HRP | Biosharp | BL002A | 1:5000 |

**Supplementary Table 3. Details of Antibody and dilution rate for immunofluorescent staining**

| Antibody | Manufacturer | Product numbers | Dilution Rate |
| --- | --- | --- | --- |
| Rabbit anti-DCX | Proteintech | 13925-1-AP | 1:200 |
| Rabbit anti-Nestin | Abcam | ab221660 | 1:500 |
| Rabbit anti-SIRT2 | Cell Signaling Technology | 12650S | 1:200 |
| Rabbit anti-MAP2 | Proteintech | 17490-1-AP | 1:500 |
| Goat anti-rabbit IgG-Cy3 | Servicebio | GB21303 | 1:200 |
| Goat anti-rabbit IgG-Alexa Flour 488 | Servicebio | GB25303 | 1:500 |

**Supplementary Table 4. Genes primers used for real time PCR analyses**

| Gene | Primer sequences |
| --- | --- |
| *Gapdh* | Forward: 5’-AGGTCGGTGTGAACGGATTTG-3’  Reverse: 5’-TGTAGACCATGTAGTTGAGGTCA-3’ |
| *Sirt2* | Forward: 5’-GCGGGTATCCCTGACTTCC-3’  Reverse: 5’-CGTGTCTATGTTCTGCGTGTAG-3’ |
