# Supplementary material for: Oligodendrocyte‐derived exosomes‐containing SIRT2 ameliorates depressive‐like behaviors and restores hippocampal neurogenesis and synaptic plasticity via the AKT/GSK‐3β pathway in depressed mice
Source: CNS Neurosci Ther. 2024 Mar 4;30(3):e14661. doi: 10.1111/cns.14661 (PMC10912796; doi:10.1111/cns.14661)
Supplement: Supplementary file 1 — Figures S1–S12 [file CNS-30-e14661-s001.zip › Supplement File 1-Figure Captions.doc]

**Supplementary Figure captions**

**Figure S1.** Full unedited gels of Figure 1C. Western blotting analysis of CD9, CD63, Alix, and MBP in TEXs and ODEXs.

**Figure S2.** Full unedited gels of Figure 2D. Western blotting analysis of SYP and PSD95 in Control, CUMS and ODEXs groups of mice.

**Figure S3.** Full unedited gels of Figure 3A. Western blotting analysis of SIRT2 and CD63 in TEXs and ODEXs.

**Figure S4.** Full unedited gels of Figure 3E. Western blotting analysis of SIRT2 in Control, CUMS and ODEXs groups of mice.

**Figure S5.** Full unedited gels of Figure 4D. Western blotting analysis of SYP and PSD95 in Control, CUMS, ODEXs and ODEXs + AK-7 groups of mice.

**Figure S6.** Full unedited gels of Figure 4K. Western blotting analysis of p-AKT, AKT, p-GSK-3β and GSK-3β in Control, CUMS, ODEXs and ODEXs + AK-7 groups of mice.

**Figure S7.** Full unedited gels of Figure 5B. Western blotting analysis of SIRT2 in vector and SIRT2 lentivirus transfected NSPCs.

**Figure S8.** Full unedited gels of Figure 5F. Western blotting analysis of SYP and PSD95 in vector and SIRT2 lentivirus transfected NSPCs.

**Figure S9.** Full unedited gels of Figure 6A. Western blotting analysis of SIRT2 in Control and ODEXs groups of NSPCs.

**Figure S10.** Full unedited gels of Figure 6E. Western blotting analysis of SYP and PSD95 in Control, ODEXs and ODEXs + AK-7 groups of NSPCs.

**Figure S11.** Full unedited gels of Figure 6H. Immunoprecipitation analysis of acetylated AKT and western blotting analysis of SIRT2 in Control, ODEXs and ODEXs + AK-7 groups of NSPCs.

**Figure S12.** Full unedited gels of Figure 6J. Western blotting analysis of p-AKT, AKT, p-GSK-3β and GSK-3β in Control, ODEXs and ODEXs + AK-7 groups of NSPCs.
